# The Italians in the Time of Coronavirus: Psychosocial Aspects of the Unexpected COVID-19 Pandemic

**DOI:** 10.3389/fpsyt.2021.551924

**Published:** 2021-03-29

**Authors:** Francesca Favieri, Giuseppe Forte, Renata Tambelli, Maria Casagrande

**Affiliations:** ^1^Department of Psychology, Sapienza University of Rome, Rome, Italy; ^2^Department of Dynamic and Clinical Psychology and Health Studies, Sapienza University of Rome, Rome, Italy

**Keywords:** COVID-19, pandemic, coronavirus, psychological aspects, PGWBI, psychological well-being, PTSD

## Abstract

**Background:** The COVID-19 pandemic is a worldwide public health emergency that forced the Italian Government to deliberate unprecedented actions, including quarantine, with a relevant impact on the population. The present study is one of the first Italian nationwide survey within the first period of the COVID-19 outbreak aimed to understand the social and psychological impact of the COVID-19 outbreak.

**Methods:** An online survey collected information on sociodemographic data, history of direct or indirect contact with COVID-19, and other information concerning the COVID-19 emergency. The General Psychological Well-Being Index and a modified version of the PTSD Checklist for DSM-5, focused on the COVID-19 experience, assessed the respondents' general psychological condition.

**Results:** Of 1,639 respondents equally distributed in the Italian territory, 5.1% reported PTSD symptomatology, and 48.2% evidenced lower psychological well-being linked to COVID-19 diffusion. Lower psychological well-being was significantly higher in women, younger than 50 years, and with health risk factors. Lower psychological well-being was also detected in individuals who did not know if they were infected, who have had direct exposure or were uncertain about their exposure to COVID-19, or who knew infected people. Regarding the social and behavioral consequences, respondents perceived worsening in demographic, economic, social, and relational conditions. Moreover, they reported increased film viewing, cookhouse time, social media use, and decreased physical activity.

**Conclusion:** The COVID-19 pandemic appears to be a risk factor for psychological diseases in the Italian population, as previously reported in the Chinese people. About half of the respondents reported a significant psychological impact. Moreover, we confirmed the role of restraining measures that led to modify lifestyles, social perception, and confidence in the institutions. These results underline the need for further studies aimed to develop psychological interventions to minimize the consequences of the COVID-19 pandemic.

## Introduction

Since December 2019, several world places have gradually experienced an outbreak of pneumonia epidemic caused by the 2019 novel coronavirus (2019-nCoV, later named SARS-CoV-2, and then COVID-19) (1). The COVID-19 outbreak was defined as a pandemic by the World Health Organization (WHO) (1). The governments of many states immediately focused attention on the best strategies to reduce the virus diffusion and the number of victims.

In Italy, since the first case of COVID-19 (February 20, 2020), a rapid spread of the contagions was reported in the first weeks of March. This condition resulted in the Italian Government's deliberation of unprecedented actions aimed to reduce the diffusion of the virus, in line with the measures already adopted in China. Since March 10, a lockdown was requested for the Italian population. This measure included avoiding gathering and requiring to maintain the social distance of at least 1 m, limiting the number of people in public places, going out to work only if the physical presence was essential, and going out of one's own home if it was strictly necessary. Moreover, the blocking of all unnecessary economic activities (e.g., gyms, restaurants, and beauty centers) was imposed. For the first time since the end of the Second World War, the Italian population is facing a reduction in freedom of movement and a severe economic and job crisis that adds to the uncertainty linked to the increase in COVID-19 cases and victims. As of March 30, 2020, the pandemic had caused 12,428 deaths out of 105,792 confirmed cases in Italy (2). Despite the obvious benefits of the extreme social distancing measures adopted in countries such as Italy, the spread of COVID-19 is still unstoppable worldwide.

One of the main features that impact psychological well-being is the restriction of freedom of movement connected to social isolation. Previous studies on several epidemics, such as HIV/AIDS diffusion, the SARS and H1N1 pandemic, the Ebola virus, and the Zika virus, have underlined psychological consequences not only on individuals affected by these diseases but also on the non-infected community because they involve different levels of social life (3–6). Hence, both the sudden outbreak of a new and unknown virus and the measures adopted to decrease its spread have had a strong impact on the quality of life and the population's psychological well-being. Accordingly, a recent review suggested that the psychological impact of quarantine and social distancing is wide ranging, substantial, and can be long lasting. It includes anxiety and mood disorders, psychological distress and post-traumatic symptomatology, sleep disturbance, and other psychopathological conditions that negatively impact general psychological well-being and quality of life (7). However, although there are similarities with previous epidemic outbreaks and other diseases, the COVID-19 pandemic has some peculiarities, such as its rapid global spread, its high social and mass-media impact, the high uncertainty due to its origin and its consequences on global health, and the extreme measures taken on a large scale, which make it different from previous cases and underline its scientific relevance in understanding the impact of such kind of event, also on a psychological level.

Starting from the first weeks and over time, some studies have proposed investigating the psychological impact of the COVID-19 pandemic in the first phases of its spread [e.g., 5, 6, 8]. However, most of the first research focused on identifying the epidemiology and clinical characteristics of patients infected by the virus (8, 9) and the challenges for the health systems and the national and international institutions (10). More recent studies analyzed the psychological effects of this emergency in more detail in Italian samples (11–13).

The present study is part of a first nationwide, large-scale survey conducted in the Italian population within the first and more tumultuous weeks of the COVID-19 outbreak (March 2020), focused on assessing the general psychological well-being of the Italian population and the perceptions about the impact of this experience on the Italians' life. Our goal is to provide a photograph of the Italian condition in the first weeks of the restrictive measures related to the period immediately following the promulgation of the “I stay home” decree determined by the broad and severe diffusion of the COVID-19 in Italy.

## Methods

### Setting and Participants

A cross-sectional design to assess the public response during the epidemic of COVID-19 was adopted. We used an anonymous online survey disseminated to platforms and social media. Due to the current research aim, being at least 18 years old was the only inclusion criterion. The 97.03% of the total respondents (1,689) that started the questionnaires completed the whole survey (1,639) and were considered for the statistical analysis. The main demographic characteristics of the sample are shown in Table 1.

**Table 1:** Demographic characteristics of the sample and information about COVID-19.

***n* (%)**	**Overall sample (*N* = 1,639)**	**High general psychological well-being (*N* = 947)**	**Low general psychological well-being (*N* = 692)**	*****χ^2^*****	***P***
Gender				73.96	0.0001
Man	394 (24.0)	301 (76.4)	93 (23.6)		
Woman	1.242 (75.8)	644 (51.9)	598 (48.1)		
Other	3 (0.2)	2 (66.7)	1 (33.3)		
Age				8.23	0.01
18–29 years old	1.088 (66.4)	617 (56.7)	471 (43.3)		
30–49 years old	353 (21.5)	197 (55.8)	156 (44.2)		
>50 years old	198 (12.1)	133(67.2)	65 (32.8)		
Education				13.72	0.003
Until middle school	71 (4.3)	48 (67.7)	23 (32.4)		
High school	808 (49.3)	452 (55.9)	356 (44.1)		
Graduate and post-graduate					
Health care	221 (13.5)	149 (67.4)	72 (32.6)		
Other	539 (32.9)	298 (55.3)	241 (44.7)		
Occupation				6.22	0.18
Student	764 (46.6)	425 (55.6)	339 (44.4)		
Employed	506 (30.9)	306 (60.5)	200 (39.5)		
Unemployed	187 (11.4)	101 (54.0)	86 (46.0)		
Self-employed	155 (9.5)	98 (63.2)	57 (36.8)		
Retired	27 (1.6)	17 (63.0)	10 (37.0)		
Health risk factor^*^				25.03	0.0001
No	1.219 (74.4)	748 (61.4)	471 (38.6)		
Yes	420 (25.6)	199 (47.4)	221 (52.6)		
Italian territorial areas				1.48	0.47
North Italy	496 (30.3)	282 (56.9)	214 (43.1)		
Center Italy	535 (32.6)	302 (56.4)	233 (43.6)		
South Italy	608 (37.1)	363 (59.7)	245 (40.3)		
Average number of inhabitants, n (%)				3.81	0.28
<2,000	85 (5.2)	49 (57.6)	36 (42.4)		
2,000–10,000	335 (20.4)	178 (53.1)	157 (46.9)		
10,000–100,000	644 (39.3)	379 (58.9)	265 (41.1)		
>100,000	575 (35.1)	341 (59.3)	234 (40.7)		
Number of usual daily interaction (no. of people)				7.63	0.04
<10	663 (40.5)	359 (54.1)	304 (45.9)		
10–50	787 (48.9)	477 (60.6)	310 (39.4)		
51–100	147 (9.0)	83 (56.5)	64 (43.5)		
>100	42 (2.6)	28 (66.7)	14 (33.3)		
Quarantine experience				4.62	0.59
Alone	166 (10.1)	98 (58.7)	69 (41.3)		
Family members	1.153 (70.4)	666 (57.8)	487 (42.2)		
Roommates	97 (5.9)	46 (52.3)	42 (47.7)		
Partner	217 (13.2)	127 (58.5)	90 (41.5)		
Co-workers	6 (0.4)	2 (33.3)	4 (66.7)		
Occupational status during COVID-19 emergency				13.40	0.009
Unemployed	787 (48.0)	436 (55.4)	351 (44.6)		
Keep on working out	166 (10.1)	98 (59.0)	68 (41.0)		
Smart-working	289 (17.6)	175 (60.6)	114 (39.4)		
Not-working					
Economic problem	150 (9.2)	75 (50.0)	75 (50.0)		
No economic problem	247 (15.1)	163 (66.0)	84 (34.0)		
Infected by COVID-19				7.57	0.02
Yes	6 (0.4)	2 (33.3)	4 (66.7)		
No	1.189 (72.5)	710 (59.7)	479 (40.3)		
Do not know	444 (27.1)	235 (52.9)	209 (47.1)		
Direct contact with people infected by COVID-19				11.93	0.002
Yes	32 (2.0)	12 (37.5)	20 (62.5)		
No	991 (60.4)	601 (60.6)	390 (39.4)		
Do not know	616 (37.6)	334 (54.2)	282 (45.8)		
Knowledge of people infected by COVID-19				12.99	0.01
Acquaintance	231 (14.1)	132 (57.1)	99 (42.9)		
Co-worker	33 (2.0)	19 (57.6)	14 (42.4)		
Friend	87 (5.3)	37 (42.5)	50 (57.5)		
Family	83 (2.6)	19 (44.2)	24 (55.8)		
No	1.245 (76.0)	740 (59.4)	505 (40.6)		
Knowledge of people in ICU for COVID-19				6.97	0.13
Acquaintance	87 (5.3)	49 (56.3)	38 (43.7)		
Co-worker	9 (0.5)	4 (44.4)	5 (55.6)		
Friend	29 (1.8)	11 (37.9)	18 (62.1)		
Family	12 (0.7)	5 (41.7)	7 (58.3)		
No	1.502 (92.0)	878 (58.5)	624 (41.5)		
Knowledge of people died for COVID-19				6.75	0.08
Acquaintance	68 (4.1)	34 (50.0)	34 (50.0)		
Co-worker	-	-	-		
Friend	8 (0.5)	2 (25.0)	6 (75.0)		
Family	10 (0.6)	4 (40.0)	6 (60.0)		
No	1.553 (95.8)	907(58.4)	646 (41.6)		
Awareness of the emergency state				3.62	0.30
Before the “I Stay at home” decree	1.332 (81.3)	756 (56.8)	576 (43.2)		
After the “I Stay at home” decree	301 (18.4)	187 (62.1)	114 (37.9)		
We are not in emergency state	6 (0.4)	4 (66.7)	2 (33.3)		
Confidence in the measures adopted by the Italian Government				15.18	0.0005
Yes	962 (58.7)	594 (61.7)	368 (38.3)		
No	448 (23.3)	236 (52.7)	212 (47.3)		
Do not know	229 (14.0)	117 (51.1)	112 (48.9)		

The analyses refer to the comparison between people with high and low general psychological well-being.

* *Risk factors for the COVID-19 include hypertension, diabetes, cardiovascular and pulmonary diseases, immunodepression, oncological pathologies, kidney disorders, or other medical conditions also in comorbidities*.

### Procedure and Survey Development

As the Italian Government recommended to minimize face-to-face interactions, participants completed the questionnaires through an online survey platform (KoboToolbox). Expedited ethics approval was obtained from the Ethics Committee of the Department of Dynamic and Clinical Psychology of “Sapienza” University of Rome (protocol number: 0000266). The study conformed to the principles of the Declaration of Helsinki. All respondents provided electronic informed consent before starting the investigation. Data refers to the period from March 18 to 25, 2020. The structured survey consisted of questions that covered several areas and took ~30 min to complete. After a demographic questionnaire, participants responded to items assessing the knowledge and perceptions of COVID-19 diffusion and the government measures adopted to contain it; then, questionnaires to evaluate psychological aspects were administered. Participants could withdraw from the survey without providing any justification, and no data were saved. Only data with a complete set of responses were considered.

### Outcomes

Sociodemographic data were collected on gender, age, education, current location, employment status, and the number of usual day interactions. The social impact of COVID-19 was measured, including quarantine experience, level of confidence in State Institution, information about COVID-19, the trend of new cases and deaths, and previsions on the potential end of the infection. Respondents were required to indicate their source of information and their confidence with it. Concerns about COVID-19 variables included self and other family members that had or could contract the COVID-19 virus. The psychological impact of COVID-19 was measured using the *Psychological General Well-Being questionnaire (PGWB)* (14) and the *Post-Traumatic Stress Disorder Related to COVID-19*.

The *Psychological General Well-Being questionnaire* (PGWB) (14) was adopted to measure subjective general psychological well-being. The PGWB consists of 22 items on six-point Likert scales, divided into six dimensions: Anxiety, Depressed mood, Positive well-being, Self-control, General health, and Vitality. A global score and measures for each dimension are calculated, with higher scores indicating greater well-being in subscales and global scores. In our study, scores higher than 60 indicate adequate psychological general well-being (15).

The *Post-Traumatic Stress Disorder Related to COVID-19* (COVID-19-PTSD; Modified version of PTSD Checklist for DSM-5; PCL-5) (16, 17) is a self-report measure designed *ad hoc* to assess specific symptoms consequent to the COVID-19, similar to PTSD symptoms, according to the DSM-5 criteria. The questionnaire includes 19 items structured in five-point Likert scales, from 0 (not at all) to 4 (extremely). To test the psychometric quality of this questionnaire, data were collected in an independent subsample (*n* = 300, 150 women; mean age: 26.22 ± 1.27). The principal component analysis indicated one factorial structure, including 19 items that explain 49% of the variance. Then, confirmatory factor analysis confirmed a mono-factorial structure with 19 items with good model fits and adequate reliability (CFI = 0.80, SRMR = 0.06, χ2/ = 871.45 with *p* <0.001; Cronbach's α: 0.94). In this study, scores higher than the mean of the sample plus 1.5 standard deviations were indicative of higher PTSD symptomatology.

### Statistical Analysis

Descriptive statistics were calculated for sociodemographic characteristics, and social and psychological variables were collected. Prevalence data (number and percentage) for each dimension assessed were reported. The scores of the PWBI subscales and COVID-19-PTSD test were expressed as means and standard deviations. T-Student was used to compare our sample data with normative data. Chi-square test (χ^2^) was used to compare the differences in prevalence between groups with high general psychological well-being and low general psychological well-being. Logistic regression and correlation models were performed to explore potential influence factors for psychological well-being and PTSD symptomatology during the COVID-19 epidemic. Odds ratio (OR) and 95% confidence interval (95% CI) were obtained from logistic regression models. *P*-values of < 0.05 were considered statistically significant (two-tailed test). All data were analyzed using the Statistical Package for Social Sciences (SPSS) version 24.0 (IBM SPSS Statistics, New York, United States) and Statistica 10.0.

## Results

### Demographic Characteristics

Sociodemographic characteristics are presented in Table 1.

One thousand six hundred and thirty-nine respondents completed the questionnaires (completion rate: 97.04%). The mean age of the respondents was 30.37 (SD: 12.14) years. The majority of the respondents were women (75.8%), aged from 19 to 30 years (66.4%), students (46.6%), or employees (30.9%). Respondents were equally distributed in Italian territorial areas, 30.3% in the North, 32.6% in the Center, and 37.1% in the South of Italy. Most respondents lived in a city with over 10,000 inhabitants (74.5%) and declared to generally have from 10 to 50 daily interactions with others (48.9%). In most cases, the quarantine experience was shared with members of the family (70.4%). The majority of the respondents reported that they had perceived the emergency state before the restrictive actions taken by the government with the “I stay at home” decree (81.3%).

Considering the history of contacts with confirmed and suspected cases of COVID-19, overall, 2.0% of the respondents have been in direct contact with an individual with suspected COVID-19; 8.3 and 5.2% reported, respectively, to know a person who was currently an inpatient in an intensive care unit or died because of the COVID-19 infection.

### Social Aspects

The measures taken by the government were perceived by more than half of the respondents as adequate (58.7%) (see Table 1). Considering the confidence in the source of information about COVID-19, the respondents reported being fairly confident of health information available by Civil Protection (49.4%), government (49.4%), general practitioner (33.7%), and scientific journal (36.1%). Confidence in social media information (35.8%) was relatively low (see Table 2).

**Table 2:** Confidence of the responders on specific sources of information during the COVID-19 emergency.

***n (%)***	**Extremely**	**Quite a bit**	**Indifferent**	**A little bit**	**Not at all**
Italian government	485 (29.6)	810 (49.5)	125 (7.6)	176 (10.7)	43 (2.6)
Civil protection	666 (40.6)	739 (45.1)	111 (6.7)	105 (6.4)	18 (1.2)
General practitioner	64 (16.1)	553 (33.7)	476 (29.0)	185 (11.4)	161 (9.8)
Social media	67 (4.1)	378 (23.1)	331 (20.2)	87 (35.8)	272 (16.8)
Scientific journals	619 (37.8)	591 (36.1)	264 (16.1)	68 (4.1)	97 (5.9)

Regarding the social consequences of COVID-19 (see Table 3), the respondents perceived high improvement in the environmental condition (37.8%) and high worsening in demographic (31.0%), economic (82.6%), social (41.7%), and relationship (39.1%) conditions. Moreover, no relevant changes were perceived on identification (22.9%), politic (28.4%), and cultural (28.9%) conditions.

**Table 3:** Perception of some social conditions by responders during the COVID-19 emergency.

***n (%)***	**Significantly improved**	**Moderately improved**	**Neither improved or worst**	**Moderately worst**	**Significantly worst**	**Do not know**
Environmental condition	620 (37.8)	624 (38.1)	142 (8.7)	66 (4.0)	79 (4.8)	108 (6.6)
Cultural condition	22 (1.3)	245 (15.0)	473 (28.9)	384 (23.5)	337 (20.6)	178 (10.7)
Demographic condition	14 (0.9)	46 (2.8)	289 (17.6)	395 (24.1)	508 (31.0)	387 (23.6)
Economic condition	21 (1.3)	13 (0.8)	34 (2.1)	127 (7.7)	1.345 (82.6)	99 (6.0)
Political condition	31 (1.9)	218 (13.4)	466 (28.4)	307 (18.7)	294 (17.9)	323 (19.7)
Social condition	34 (2.1)	191 (11.6)	199 (12.1)	418 (25.5)	683 (41.7)	114 (7.0)
Relationship status	52 (3.2)	222 (13.5)	244 (14.9)	377 (23.0)	641 (39.1)	103 (6.3)
Sense of identity	67 (4.1)	239 (14.6)	376 (22.9)	257 (15.7)	306 (18.7)	394 (24.0)

Considering changes in activity during the COVID-19 emergency, respondents reported an increase in film viewing (38.1%), cookhouse time (33.4%), use of social media (39.8%), and a decrease in physical activity (28.8%) (see Table 4).

**Table 4:** Activity changes of the responders during the COVID-19 emergency.

***n (%)***	**Significantly increased**	**Moderately increased**	**Unchanged**	**Moderately decreased**	**Significantly decreased**
Book reading	267 (16.3)	387 (23.4)	823 (50.2)	49 (3.0)	116 (7.1)
Film viewing	624 (38.1)	536 (32.7)	386 (23.6)	48 (2.9)	45 (2.7)
Physical activity	186 (11.3)	280 (17.1)	448 (27.3)	253 (15.4)	472 (28.8)
Cookhouse time	429 (26.2)	547 (33.4)	33 (32.5)	66 (4.0)	64 (3.9)
Use of social media	652 (39.8)	514 (31.4)	402 (24.5)	31 (1.9)	40 (2.4)

### Psychological Aspects

The sample showed a lower mean than normative data in the PGWB questionnaire, considering all the subscales and total well-being, confirming low psychological well-being (see Table 5).

**Table 5:** Comparisons between responders' results and normative data on PGWB.

	**Survey responders**	**Normative data**	***t***	***P***
**Psychological well-being, mean (SD)**				
Anxiety	62.35 (20.97)	72.80 (19.18)	14.46	0.0001
Depressed mood	79.06 (15.77)	83.35 (16.43)	7.43	0.0001
Positive well-being	44.55 (17.23)	62.67 (18.65)	28.18	0.0001
Self-control	69.83 (18.70)	80.27 (18.80)	15.52	0.0001
General health	70.37 (15.84)	75.87 (18.47)	8.94	0.0001
Vitality	58.29 (18.80)	68.48 (18.32)	15.29	0.0001
Total well-being	62.77 (15.13)	72.86 (15.56)	18.34	0.0001

Overall, the prevalence of COVID-19-PTSD was 5.1%, with a mean total score of 19.88 (SD: 15.81), and the prevalence of lower psychological well-being was 48.2%, with a mean total well-being score of 62.77 (15.13). The prevalence of lower psychological well-being was significantly influenced by gender (X^2^ = 73.96; *p* < 0.0001), age (X^2^ = 8.23; *p* < 0.01), education (X^2^ = 13.72; *p* < 0.003), health risk factors (X^2^ = 25.03; *p* < 0.0001), number of usual daily interactions (X^2^ = 7.63; *p* = 0.04), history of infection by COVID-19 (X^2^ = 7.57; *p* = 0.02), direct contact with people infected by COVID-19 (X^2^ = 11.93; *p* = 0.002), occupational status during the COVID-19 emergency (X^2^ = 13.40; *p* = 0.009), knowledge of people infected by COVID-19 (X^2^ = 12.99; *p* = 0.01), and confidence in the measures adopted by the government (X^2^ = 15.18; *p* = 0.0005). All Chi-squared values are reported in Table 1.

Positive linear correlations were found between age and dimension of psychological well-being, except considering general health (*p* = 0.01) and PTSD symptomatology related to COVID-19 (*p* = 0.02) that reported negative correlations. Conversely, considering the number of days spent in quarantine, negative linear correlations were observed with psychological well-being and a positive one with PTSD symptomatology related to the COVID-19. The correlational matrix is shown in Table 6.

**Table 6:** Pearson's r correlations between psychological dimensions of distress, age, and time spent in quarantine.

	**Anxiety (PGWB)**	**Depression (PGWB)**	**Positive well-being (PGWB)**	**Self-control (PGWB)**	**General health (PGWB)**	**Vitality (PGWB)**	**Total well-being (PGWB)**	**PTSD symptoms**
Age	0.05	0.09	0.06	0.13	−0.10	0.08	0.10	−0.06
	*p* < 0.05	*p* < 0.0001^*^	*p* < 0.05	*p* < 0.0001	*p* < 0.01	*p* < 0.0001	*p* < 0.01	*p* < 0.05
Quarantine (no. of days)	−0.06	−0.06	−0.019	−0.04	−0.05	−0.07	−0.06	0.08
	*p* < 0.01^*^	*p* < 0.01^*^	*p* < 0.4	*p* = 0.09	*p* = 0.07	*p* < 0.01^*^	*p* < 0.01^*^	*p* < 0.01^*^

* *Bonferroni adjusted p ≤ 0.02*.

Logistic regression models showed statistical differences (Table 7). Being a woman (OR = 3.00; 95% CI = 2.31–3.88), belonging to the age groups of 18–29 (OR = 1.56; 95% CI = 1.13–2.15) and 30–49 years (OR = 1.62; 95% CI = 1.12–2.33), with a high school degree (OR = 1.63; 95% CI = 1.19–2.23) or a bachelor degree in disciplines other than healthcare (OR = 1.6; 95% CI = 1.2-2.3), and with the presence of health risk factors (OR = 1.76; 95% CI = 1.41–2.20) represent higher risk conditions for experiencing low psychological well-being. Regarding the COVID-19 outcome role on psychological well-being, respondents who were uncertain about the COVID-19 infection (OR = 1.31; 95% CI = 1.05–1.64) or who had some contacts with individuals affected by the virus (OR = 2.6; 95% CI = 1.2–0.3) were more likely to report low psychological well-being, as well as people who knew someone infected by COVID-19 (OR = 1.32; 95% CI = 1.05–1.66) or who knew people who were hospitalized in intensive care units (OR = 1.38; 95% CI = 0.97–1.96) or dead people consequent of COVID-19 infection (OR = 1.61; 95% CI = 1.04–2.49). Finally, no confidence (OR = 1.45; 95% CI = 1.15–1.81) or uncertainty (OR = 1.54; 95% CI = 1.15–2.06) about the suitability of the measures adopted by the Italian Government impacted negatively on psychological well-being (see Table 7).

**Table 7:** Results of the logistic regression.

	**PGWBI**			**PTSD symptoms**		
	**B**	**OR (95% CI)**	***P***	**B**	**OR (95% CI)**	***p***
**Gender**						
Man	Reference		Reference			
Woman	1.09	3.0 (2.3–3.9)	0.0001	1.89	6.6 (2.4–18.1)	0.0001
**Age**						
18–29 years old	0.44	1.7 (1.1–2.1)	0.01	0.76	2.1 (0.8–5.4)	0.11
30–49 years old	0.48	1.6 (1.1–2.3)	0.01	0.89	2.4 (0.9–6.6)	0.08
>50 years old	Reference			Reference		
**Education**						
Until Middle School	−0.01	1.0 (0.6–1.7)	0.97	−1.09	0.3 (0.01–2.7)	0.31
High School	0.49	1.6 (1.2–2.2)	0.01	0.18	1.2 (0.6–2.5)	0.64
**Graduate and post-graduate**						
No Health Care	0.51	1.6 (1.2–2.3)	0.01	0.46	1.6 (0.7–3.4)	0.22
Health Care	*Reference*			*Reference*		
**Occupation**						
Student	0.23	1.2 (0.6–2.8)	0.57	0.34	1.4 (0.2–10.6)	0.75
Employed	0.10	1.0 (0.4–2.3)	0.97	0.82	2.3 (0.3–17.9)	0.44
Unemployed	0.31	1.4 (0.6–3.2)	0.46	0.14	1.2 (0.1–8.8)	0.89
Retired	Reference			Reference		
**Health risk factor**						
No	Reference			Reference		
Yes	0.57	1.8 (1.4–2.2)	0.0001	0.90	2.5 (1.6–3.9)	0.0001
**Italian Territorial Areas**						
North Italy	0.11	1.1 (0.9–1.4)	0.33	0.29	1.3 (0.8–2.2)	0.28
Center Italy	0.13	1.1 (0.9–1.4)	0.26	−0.11	0.9 (0.5–1.6)	0.70
South Italy	Reference			Reference		
**Average number of inhabitants**, ***n*** **(%)**						
<2,000	Reference			Reference		
2,000–10,000	0.18	1.2 (0.7–1.9)	0.45	0.30	1.3 (0.5–3.6)	0.55
10,000–100,000	−0.50	1.0 (0.6–1.5)	0.83	−0.25	0.8 (0.3–2.1)	0.62
>100,000	0.70	0.9 (0.6–1.5)	0.77	−0.45	0.6 (0.2–1.7)	0.37
**Number of usual daily interaction (no of people)**						
<10	Reference			Reference		
10–50	−0.26	0.8 (0.6–0.9)	0.01	−0.18	0.8 (0.5–1.3)	0.45
51–100	−0.09	0.9 (0.6–1.3)	0.61	−0.17	0.8 (0.4–1.9)	0.69
>100	−0.53	0.6 (0.3–1.1)	0.11	−0.16	0.8 (0.2–3.6)	0.82
**Quarantine experience**						
No	Reference			Reference		
Yes	−0.03	1.0 (0.7–1.3)	0.75	0.21	1.2 (0.6–2.4)	0.55
**Occupational status during COVID-19 emergency**						
Unemployed	Reference			Reference		
Keep on working out	−0.15	0.9 (0.6–1.2)	0.39	−0.67	0.5 (0.2–1.3)	0.16
Smart-working	−0.21	0.8 (0.6–1.1)	0.13	−0.10	0.9 (0.5–1.6)	0.74
Not-working						
Economic problem	0.22	1.2 (0.9–1.8)	0.22	0.36	1.4 (0.7–2.8)	0.29
No economic problem	−0.45	0.6 (0.5–0.9)	0.01	0.89	0.4 (0.2–0.9)	0.05
**Infected by COVID-19**						
No	Reference					
Do not know	0.27	1.3 (1.1–1.6)	0.01	0.55	1.7 (1.1–2.7)	0.05
Yes	1.08	2.9 (0.5–16.2)	0.21	N/A	N/A	N/A
**Direct contact with people infected by COVID-19**						
No	Reference			Reference		
Do not know	0.23	1.3 (1.1–1.6)	0.01	0.42	1.5 (0.9–2.4)	0.07
Yes	0.93	2.6 (1.2–5.3)	0.01	1.20	3.3 (1.1–9.9)	0.05
**Knowledge of people infected by COVID-19**						
No	Reference			Reference		
Yes	0.29	1.3 (1.1–1.7)	0.01	0.78	2.2 (1.4–3.4)	0.0001
**Knowledge of people in ICU for COVID-19**						
No	Reference			Reference		
Yes	0.33	1.4 (0.9–1.9)	0.05	0.95	2.6 (1.4–4.7)	0.01
**Knowledge of people died for COVID-19**						
No	Reference			Reference		
Yes	0.48	1.6 (1.0–2.5)	0.05	0.85	2.3 (1.1–4.8)	0.05
**Confidence in Italian Government**						
No	0.37	1.5 (1.1–1.8)	0.001	1.09	2.9 (1.9–4.7)	0.0001
Do not know	0.43	1.5 (1.2–2.1)	0.01	−0.50	0.6 (0.2–1.6)	0.31
Yes	Reference			Reference		

Concerning PTSD symptomatology, the previous results on PGWB on women (OR = 6.6; 95% CI = 2.4–18.1), health status (OR = 2.5; 95% CI = 1.6–3.9), uncertainty about the presence of COVID-19 infection (OR = 1.7; 95% CI = 1.1–2.7), having had direct contacts with people infected by COVID-19 (OR = 3.3; 95% CI = 1.1–1.9), knowing someone infected by COVID-19 (OR = 2.2; 95% CI = 1.4–3.4), or hospitalized in intensive care units (OR = 2.6; 95% CI = 1.4–4.7), or dead people consequent to the COVID-19 infection (OR = 2.3; 95% CI = 1.1–4.8), represent high risk factors to develop PTSD symptomatology (see Table 7).

## Discussion

According to the results of this study, just a few weeks after the outbreak of the COVID-19 in Italy and a few days after the declaration of the restrictive measures, 5.1% of the respondents reported PTSD symptomatology linked to the COVID-19 diffusion, and 48.2% evidenced lower psychological well-being, characterized by anxiety and depressive symptoms, negative well-being, perception of loss control, less vitality, and lower general perceived health. These data are in line with those found by Sun and colleagues (18). The authors reported a percentage of 4.6 of PTSD symptoms in a large sample of the Chinese population (*n* = 2.091) with ages ranging from 30 to 60 years, and with some Italian studies that focused on COVID-19-related psychological distress in the Italian population (12, 13, 19, 20). In further studies, it could be interesting to compare the results obtained by Italian samples with samples from other countries (e.g., China) to check the similarities and differences on both psychological status and social and cultural changes that are COVID-19 related.

As expected, the comparison between our data with normative ones (15) on an Italian sample of healthy people suggested lower levels of general psychological well-being in Italians living this extraordinary emergency condition. These results would confirm that the stressful impact of the COVID-19 condition on psychological well-being is similar to the psychological burden caused by SARS and other virus outbreaks reported by studies in different countries (18, 21–23). These studies reported high levels of distress, anxiety, and mood disorders.

Considering sociodemographic and lifestyle information, the photograph of Italians in the coronavirus' time reported that most respondents spent social isolation at home, not quite alone but generally with other family members. The number of direct or indirect contacts with people affected by COVID-19 seems to be relatively low in the sample, especially considering the high reproductive number of the COVID-19 (24, 25). However, this result could be affected by the data collection in the early COVID-19 spread in the Italian population.

The results on the number of days spent in quarantine underlined a relationship with different aspects associated with psychological distress, such as anxiety, depression, decreasing of vitality, global well-being, and a general PTSD symptomatology. These results would confirm the effect of the restrictive measures on psychological aspects, highlighting how the higher number of days spent in quarantine can play a cumulative role in developing distress, in line with previous studies on the effect of social isolation and quarantine (26). However, further studies are needed in order to report other risk factors and hopefully implement remote delivery of psychological interventions, to control psychological distress during the first stages of the present emergency.

Generally, we evidenced interesting results on the risk factors for well-being and psychological distress in emergencies due to the COVID-19 pandemic. Women, individuals younger than 50 years, those with high school degree or a bachelor's degree in disciplines other than health care, and those who present health risk factors seem to be more likely to have low psychological well-being. Moreover, people who are sure that they have had no contact with people affected by the infection reported higher psychological well-being levels. Also, those who have had close relationships with individuals (i.e., family members and friends), who were affected more or less severely by the virus, reported low psychological general well-being, confirming a role of fear and uncertainty about the epidemic progression on the levels of psychological distress (6). Finally, no confidence or uncertainty about the suitability of the Italian Government's measures negatively impacted psychological well-being, probably also as a consequence of the changes in the lifestyles, as suggested by our results.

These findings agree with previous studies (5, 6, 18, 26). The risk of infection generates fear in people, and above all, the COVID-19-related stressors, which include economic, daily life, social, and relational stressors, appear to be associated with worse psychological well-being. It would be useful to consider these aspects in order to implement specific interventions to prevent worsening of the psychological symptoms, leading to real psychological diseases, such as posttraumatic stress disorder. Both general demographic conditions and risk factors more closely related to the COVID-19 spread seem to influence the individuals, generating high levels of distress, and in some cases, they represent a warning sign for PTSD symptomatology, as confirmed by our results.

Another aim of this study was to consider the social impact of the COVID-19 outbreak. Our findings suggest that despite the optimism that referred to an improvement of the environmental condition, respondents had a negative perception about the influence of the COVID-19 on life and social aspects. People largely perceived a moderate to drastic worsening of the economic and social conditions, although most of the respondents appeared highly confident about the information on the COVID-19 derived from state institutions as the Italian Government and the Civil Protection and even if they considered the restrictive measures taken by the government to be appropriate to the epidemic emergency. These findings agree with those reported during the H1N1 epidemic in Hong Kong (27), which showed a similar ambivalence between the general support toward the government and a sense of low confidence in its success to control the epidemic diffusion. The drastic changes due to the Italian Government measures to contain the COVID-19 were perceived as adequate for a large majority of the population during the survey, but they inevitably impacted daily life and recreational activities. The need for further risk analysis to identify what aspects of the pandemic emergency impact people, reduce the risk of psychopathological conditions that arise or persist even over the acute emergency, and represent an additional burden for the public health system is highlighted (28). This should be done in the light of the extension overtime of the state of emergency in several countries around the world (e.g., Spain, United States, and Brazil), the risk of second waves of the contagion, and the long-term consequences of the pandemic state of emergency.

The findings of this study provide the first evidence about the necessity to develop a psychological support strategy in the Italian population during and after the state of emergency, according to the suggestions of previous studies on epidemics (21, 29–33). It is crucial to prepare healthcare systems for the long-term medical and psychological consequences of this pandemic. Although further studies are needed, our results could help identify more high-risk populations for clinical diseases.

This study has some limitations. First, our design did not allow making a causal relationship, and prospective studies are needed to make causal inferences. Second, the possibility of selection bias due to the online survey should be considered, as evidenced by the oversampling of a particular population (e.g., students and women). This limit is particularly important to take into account considering the risk factor analyses, which the characteristics of the sample could influence. Another limitation is the use of self-report measures in the online survey. These instruments, especially if administered remotely, may be subjected to data collection biases. However, as reported in other studies on the COVID-19 pandemic, the adoption of an online survey represented the best solution because the social distancing measures limited data collection.

Therefore, the number of respondents with contact history with the COVID-19 was low, probably due to the data collection times. In the first days of the pandemic, the COVID-19 was not yet widespread in the Italian population, making our findings not generalizable to confirmed cases of COVID-19. Finally, it is more difficult to differentiate the influences due to the medical impact of the infection from the impact of quarantine measures, and further studies should consider it.

Our findings indicate that the COVID-19 pandemic appears to be an influential risk factor for the development of psychological diseases in the Italian population, as reported by other studies (5, 6, 18, 26) also on Italian samples (12, 19, 20), and accordingly with other studies on epidemic and quarantine conditions (7, 21, 34). The COVID-19 outbreak has substantially changed lifestyles, social perception, and institutions' confidence in the Italian and worldwide population, and it appears to have significant psychological consequences. Despite some limitations, this study provides information on the initial psychological, social, and lifestyle responses during the outbreak of COVID-19 in Italy. Moreover, it provides an interesting point of view suggesting how future studies should distinguish between the effects directly resulting from the spread of the virus, and therefore closely related to the fears of contagion, and those caused by changes in people's lifestyle, due to more or less severe containment measures taken by governments. Our main goal was to demonstrate that the psychological and social impact of this outbreak cannot be minimized. According to our results, we propose considering the psychological, social, relational, and behavioral consequences of these exceptional events in interventions aimed at improving or preventing psychological distress and their impact on the general population's quality of life.

## Data Availability Statement

The raw data supporting the conclusions of this article will be made available by the authors, without undue reservation.

## Ethics Statement

The studies involving human participants were reviewed and approved by Ethics Committee of the Department of Dynamic and Clinical Psychology, “Sapienza” University of Rome. The patients/participants provided their written informed consent to participate in this study.

## Author Contributions

MC, FF, and GF had the idea for and designed the study and had full access to all of the data in the study and take responsibility for the integrity of the data and the accuracy of the data analysis, and drafted the paper. FF and GF did the analysis. MC, FF, GF, and RT collected the data. All authors agreed to be accountable for all aspects of the work in ensuring that questions related to the accuracy or integrity of any part of the work are appropriately investigated and resolved. All authors critically revised the manuscript for important intellectual content and gave final approval for the version to be published.

## Conflict of Interest

The authors declare that the research was conducted in the absence of any commercial or financial relationships that could be construed as a potential conflict of interest. The reviewer CL declared a shared affiliation, though no other collaboration, with the authors FF, MC, GF, and RT to the handling editor.
